# Gallium Protoporphyrin Liquid Crystalline Lipid Nanoparticles: A Third-Generation Photosensitizer against *Pseudomonas aeruginosa* Biofilms

**DOI:** 10.3390/pharmaceutics14102124

**Published:** 2022-10-06

**Authors:** Muhammed Awad, Timothy J. Barnes, Nicky Thomas, Paul Joyce, Clive A. Prestidge

**Affiliations:** 1 Centre for Pharmaceutical Innovation, University of South Australia, Clinical and Health Sciences, Adelaide 5000, Australia; 2 Basil Hetzel Institute for Translational Health Research, Woodville 5011, Australia

**Keywords:** antimicrobial, biofilm, photodynamic therapy, liquid crystal nanoparticles, cubosomes

## Abstract

The looming antimicrobial resistance pandemic has encouraged the investigation of antimicrobial photodynamic therapy (aPDT) as a promising technology to combat recalcitrant bacterial infections caused by antibiotic resistant strains. Here, we report on the optimization and effective application of gallium protoporphyrin liquid crystalline lipid nanoparticles (GaPP-LCNP) as a photosensitizer for aPDT against the Gram-negative bacteria *P. aeruginosa* in both planktonic and biofilm modes of growth. LCNP significantly enhanced the performance of GaPP as photosensitizer by two-fold, which was correlated with higher antibacterial activity, reducing the viability of planktonic *P. aeruginosa* by 7 log_10_ using 0.8 µM GaPP-LCNP and a light dose of 17 J.cm^−2^. Importantly, GaPP-LCNP also reduced the viability of biofilms by 6 log_10_ at relatively low light dose of 34.2 J.cm^−2^ using only 3 µM GaPP-LCNP. The high antibiofilm activity of GaPP-LCNP at low GaPP-LCNP dose indicated the high efficiency and safety profile of GaPP-LCNP as a promising platform for photodynamic inactivation of recalcitrant infections.

## 1. Introduction

Antimicrobial photodynamic therapy (aPDT) has evolved as a treatment paradigm to control the looming number of multi-drug resistant microbes [1]. Unlike antibiotics, aPDT attacks multiple targets of microbial cells, limiting their ability to develop resistance [2]. The concept of aPDT is to utilize reactive oxygen species (ROS) generated from the interaction between visible light and nontoxic dyes, termed photosensitizers, to inactivate target cells [1]. The photosensitization process is akin to luminescence, where the electrons of photosensitizers transition from the ground state to excited states upon light illumination. These electrons undergo intersystem crossing, where the released energy can be transferred to molecular oxygen, generating highly reactive singlet oxygen (^1^O_2_), or undergo chain reactions, generating other ROS, e.g., hydroxyl and superoxide radicals [3].

Since photosensitizers are the cornerstone in PDT, various studies have been devoted to promoting ROS production and enhancing their bioavailability [4]. Two generations of photosensitizers have been utilized for the treatment of cancer and other vascular disorders [5]. The first generation of photosensitizers suffered from extreme hydrophobicity, low singlet oxygen quantum yield, low bioavailability, and dark cytotoxicity. Although the second generation demonstrated lower phototoxicity and higher singlet oxygen quantum yield, it still suffered poor water solubility and low selectivity to target cells [6]. To overcome these limitations a third generation of photosensitizers has evolved by loading existing photosensitizers into smart nanocarriers that can enhance their bioavailability and targeting of cancer and microbial cells [7].

Recently, gallium protoporphyrin (GaPP) has been proposed as a photosensitizer against *S. aureus* [8,9]. GaPP structural similarity with heme enables its superior uptake by *S. aureus*, compared to other porphyrins through heme acquisition pathways [9,10]. This uptake mechanism potentiates its antibacterial activity against *S. aureus* as iron mimetic agent in the dark and as photosensitizer upon light activation [8]. However, not all bacterial species are equally sensitive to GaPP as heme mimetic [10]. Bacteria have multiple approaches to acquire iron from surrounding media, such as (i) utilizing the iron in heme through hemophores, a special pathway to transport heme through cells, e.g., *S. aureus* [10,11]; (ii) the production of low molecular weight proteins termed siderophores that can acquire iron from surrounding media and transfer it into cells, e.g., *Pseudomonas aeruginosa* [12]; and (iii) the active transport of iron through cytoplasmic membrane [10]. 

The versatile approaches to acquiring iron by different bacterial species and the hydrophobicity of GaPP lower its potential as a photosensitizer for antimicrobial applications. Therefore, loading GaPP within smart nanoformulation is hypothesized to improve its bioavailability and antimicrobial activity against a wide range of microbes. Previously we have demonstrated the positive impact of liquid crystal lipid nanoparticles (LCNP) on the photodynamic activity of GaPP. Through optimizing GaPP-LCNP formulation, a significant improvement in the capabilities of GaPP as an iron mimetic agent and photosensitizer was evidenced by enhanced delivery of GaPP into *S. aureus* biofilms and higher ^1^O_2_ quantum yield upon light activation [8].

Therefore, investigating the antimicrobial activity of GaPP-LCNP against a more challenging biological target, such as *Pseudomonas aeruginosa*, is required to test its broader application as photosensitizer in aPDT. *P. aeruginosa* is an opportunistic Gram-negative pathogen associated with many chronic infections, such as chronic wounds and cystic fibrosis [13]. It is notorious for its multidrug resistance, including intrinsic resistance and biofilm formation [14]. The intrinsic resistance is mainly due to the outer membrane that limits the penetration of xenobiotics to the cytoplasm and efflux pumps that expel antibiotics out of the bacterial cells [3,13]. When in biofilms, *P. aeruginosa* live as aggregated colonies embedded within extracellular polymeric substances (EPS) [15], and the complex nature of EPS allows *P. aeruginosa* to escape host immune response and hinders antibiotics reaching the embedded cells [13].

The multi-resistance of *P. aeruginosa* to antimicrobials, including GaPP, in regular testing media, MIC > 128 µg/mL [10], make it an excellent candidate to test the potential of GaPP-LCNP as a third-generation photosensitizer. Herein, we report the impact of LCNP on GaPP interaction with light, optimize different parameters affecting the antimicrobial photodynamic activity of GaPP-LCNP against *P. aeruginosa* both in planktonic cultures and biofilm, and shed light on the importance of dose optimization in aPDT to achieve maximum antimicrobial activity. 

## 2. Materials and Methods

### 2.1. Materials

Myverol 18–92 K (product number: 4552180, containing 95% unsaturated glycerol monooleate (GMO)) was obtained from DKSH Performance Materials Australia as a donation, gallium protoporphyrin was purchased from Frontier Scientific (Logan, UT, USA), Luria Bertani (LB) media was purchased from (Thermo Fisher Scientific Australia Pty Ltd., Scoresby, VIC, Australia). Pluronic F 127 and methanol were obtained from Sigma Aldrich.

### 2.2. Fabrication of Liquid Lipid Crystal Nanoparticles (LCNP)

LCNP was prepared using the hydrotrope dilution method, as previously described [8,16]. Briefly, glycerol monooleate (15 mg) were mixed with Pluronic F127 in powder form (3 mg) and propylene glycol (0.26 g) via vortexing for 2 min in a 15 mL glass vial to form a homogenous gel, followed by the addition of 0.5 mL methanolic solution of gallium protoporphyrin (GaPP) (1.5 mM). An amount of 3 mL methanol was added to ascertain the solubilization of the mixture. A stream of nitrogen gas was used to obtain a dry film of GaPP/lipid. The dry film was dispersed in Milli-Q water to form a nanoparticle dispersion with a final volume of 5 mL. Blank was prepared similarly without the addition of GaPP.

### 2.3. Physicochemical Characterization of LCNP

#### 2.3.1. Dynamic Light Scattering

Three independent preparations were used for the determination of the average particle diameter (Z-average) and polydispersity index (PDI). Briefly, LCNP samples were diluted 1:100 in 1 mM NaCl, and the results were recorded using a Zetasizer Nano ZS (Malvern, Worcestershire, UK).

#### 2.3.2. Nanoparticle Tracking Analysis

The particles size, reported as mean diameter ± SD, was recorded using Nanosight NS300 equipped with blue (405 nm) laser. LCNP samples were diluted 1:1000 in Milli-Q water and measured in triplicates at room temperature. The particles motion was recorded using sCMOS camera and data was analyzed using analysis software (NTA 3.4 Build 3.4.003, Worcestershire, UK).

#### 2.3.3. Cryogenic Transmission Electron Microscopy

The morphology of GaPP-LCNP was captured using Glacios 200kV Cryo-TEM (Thermo Fisher Scientific^TM^). Briefly, 5 μL of GaPP-LCNP sample was applied to 300 mesh copper grids glow discharged for 30 S. A mixture of liquid ethane/propane was used for sample vitrification, and samples were kept at –180 °C during observation. Micrographs were recorded using NANOSPRT15 camera (Thermo Fisher Scientific) operating microscope at 120 kV under a bright field.

### 2.4. GaPP Loading and Entrapment Efficiency

The concentration of GaPP loaded in LCNP was determined using GaPP fluorescence at 585 nm, following excitation at 405 nm using Fluostar^®^ Omega microplate reader. A linear calibration curve was obtained in the range between (0.3–3 µM) with correlation coefficient (*r*) of 0.9998. To determine entrapped GaPP concentration, nanoparticle dispersion in water was centrifuged for 10 min at 31120 g; the unentrapped GaPP precipitated, while the supernatant containing nanoparticles was dissolved in methanol and the concentration was quantified from the corresponding calibration curve. GaPP loading percentage (DL%) and entrapment efficiency (EE%) were calculated using the following equations.

EE% = Amount of entrapped GaPP/amount of added GaPP × 100

DL% = Amount of entrapped GaPP/amount of GMO + added GaPP × 100

### 2.5. Spectroscopic Studies

Absorption spectra of GaPP and GaPP-LCNP were recorded on an Evolution ^TM^ 201/220 UV-Vis spectrophotometer. GaPP-LCNP dispersion was diluted in MQ water to reach final GaPP concentration of 1.5 µM, while unformulated GaPP was dissolved in DMSO followed by dilution in MQ water to reach a similar GaPP concentration of 1.5 µM and a final DMSO concentration of 1% V/V. Fluorescence intensities of GaPP and GaPP-LCNP for photobleaching study were recorded using Fluostar^®^ omega microplate reader.

### 2.6. Antimicrobial Photodynamic Activity

#### 2.6.1. Light Source

The light source used is a mounted LED at 405 nm wavelength (M405L4) [8]. An aspheric condenser lens, Ø1”, *f* = 16 mm, NA=0.79, ARC: 350–700 nm, was attached to the mounted LED using an SM1 Lens Tube, 1.00” thread depth to collimate the light beam to illuminate an area with a diameter of 2 cm, sufficient to illuminate four wells at time. A T-Cube LED Driver, 1200 mA Max Drive Current (LEDD1B) was used to operate the mounted LED and to control the output power. The output power was monitored using a PM100USB power meter connected to a S302C thermal sensor head, all purchased from Thorlabs (Newton, NJ, USA).

#### 2.6.2. Antimicrobial Activity against Planktonic Culture

The antibacterial photodynamic activity of GaPP and GaPP-LCNP was evaluated against *P*. *aeruginosa* (PAO1) in planktonic culture, as previously reported [17,18] with appropriate modifications. Briefly, an overnight culture of *P*. *aeruginosa* in LB broth was adjusted to 0.5 McFarland and 100 µL of bacterial suspension were mixed with 100 µL of either GaPP solution dissolved in PBS containing 1% DMSO or GaPP-LCNP in a black 96 well plate, a final GaPP concentration of 0.8 µM, for 30 min in the dark under mechanical shaking. Bacterial suspension was irradiated with blue light at 405 nm [8], irradiance (0.057 W/cm^2^) for 5 min, final energy fluence of 17.2 J/cm^2^. Dark controls were treated similarly without light illumination. The viability following treatment was determined by enumeration of colony forming units (CFU) on LB agar after serial dilution in saline.

#### 2.6.3. Antimicrobial Activity against Biofilms

The antibiofilm activity of GaPP-LCNP was evaluated against *P. aeruginosa* biofilms, using our previously reported protocol [8]. Briefly, overnight culture of *P. aeruginosa* in LB was adjusted to 0.5 McFarland, followed by 1:100 dilution in LB. A 100 µL of the diluted culture were transferred to a 96 well plate and incubated for 24 h in a static condition. Following incubation, biofilms were washed twice with saline, then 100 µL of blank LCNP, GaPP-LCNP, or unformulated GaPP were added to each well and incubated for 2 h in the dark. Photoactivation was conducted for 10 min, at energy fluence of 34.2 J/cm^2^. Biofilms were extracted from the wells by scraping with sterile pipette tips. This was followed by serial dilution in saline and plating on LB agar for CFU enumeration to assess the bacterial viability.

#### 2.6.4. Live-Dead Viability Assay

*P. aeruginosa* biofilms were established on 8 well slide chamber, as previously described [8]. After 24 h incubation time, biofilms were washed to remove unattached cells, then 200 µL of GaPP-LCNP or GaPP solution (3 µM) were added to each well in duplicates and incubated in the dark for 2 h. Two wells were illuminated at 34.2 J/cm^2^, while dark controls were protected from light using aluminum foil. Following photoactivation, treatments were removed and biofilms washed; then, live/dead stain assay protocol was followed [19] to determine the viability of biofilms. The chambers were removed, and the viability of attached biofilms was evaluated under 10 x magnification objective lens using confocal microscopy (LSM800, Zeiss, Oberkochen, Germany).

#### 2.6.5. Scanning Electron Microscopy (SEM) Images

A single colony of *P. aeruginosa* PAO1 was inoculated in LB broth under shaking for 18 h. Inoculum was adjusted to 0.5 McFarland, followed by 1:100 dilution in LB. An amount of 1 mL of bacterial suspension was carefully added to sterile coverslips placed in 24 well plate and incubated statically for 24 h at 37 °C. Biofilms were washed twice with PBS and 1 mL of GaPP-LCNP (3 µM) was added and after 2 h incubation in the dark wells were illuminated with 34 J/cm^2^ blue light. Treatment was removed and biofilms washed twice with PBS to remove unattached cells. Biofilms were placed in EM fixative overnight, washed twice with PBS+ 4% sucrose, then post-fixed in 2% osmium tetroxide for 1 h, followed by a series of dehydrations using 70%, 90%, and 100% ethanol. Further dehydration was conducted by using hexamethyldisilazane (HMDS): ethanol (100%) 1:1. HMDS was removed, and biofilms allowed to dry before mounting and coating with a platinum layer of 5 nm. Micrographs were recorded using Quanta 450 FEG Scanning Electron Microscope (FEI, Netherlands).

## 3. Results and Discussion

### 3.1. Physicochemical Characterization of GaPP-LCNP

**Figure 1:**
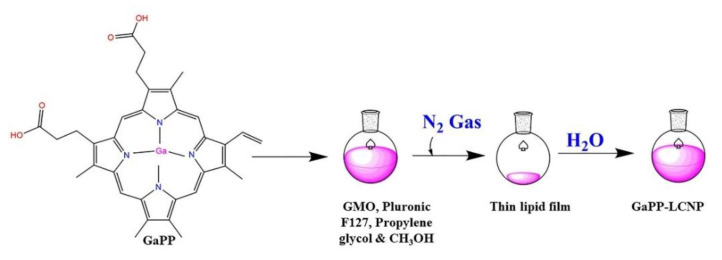
Fabrication scheme of GaPP-LCNP using hydrotrope dilution method. GaPP dissolved in methanol is mixed with glycerol monooleate (GMO), Pluronic F127, and propylene glycol; methanol is evaporated under N_2_ gas, and the lipid film is dispersed using hydrotrope dilution method. GaPP dissolved in methanol is mixed with glycerol monooleate (GMO), Pluronic F127, and propylene glycol; methanol is evaporated under N_2_ gas, and the lipid film is dispersed using MQ water.

The main goal of utilizing nanoparticles in the field of aPDT is to improve the biological activity of hydrophobic photosensitizers, such as GaPP, via enhancing their solubility in biological media [3,20]. In this study, LCNP were fabricated using hydrotrope dilution method, as shown in Figure 1 which is known to yield highly monodisperse nanoparticles with a diameter less than 200 nm [16]. LCNP successfully solubilized GaPP in aqueous solutions via entrapping GaPP molecules within the lipid bilayer with an entrapment efficiency of 98% ± 3 and GaPP loading of 3.3 ± 0.3 *w/w*% Table 1.

The stability and monodispersity of nanoparticles strongly correlate with the photodynamic activity [8]. We previously reported the photodynamic activity of GaPP-LCNP at different GaPP:lipid molar ratios and the highest photodynamic activity was recorded with 1:2 GaPP:GMO [8]. Increasing GaPP concentration did not enhance the photodynamic activity rather a steep decline in singlet oxygen quantum yield (ɸ_∆_) from 0.72 to 0.33 was reported [8]. The decline in ɸ_∆_ was correlated with lower entrapment efficiency 72% ± 5.2 and lower stability of LCNP as GaPP molecules precipitated on standing and the polydispersity index increased from 0.21 to 0.47 akin to previous reports with curcumin [21]. Therefore, we continued our characterization on GaPP-LCNP formulation, which showed the highest stability. 

**Figure 2:**
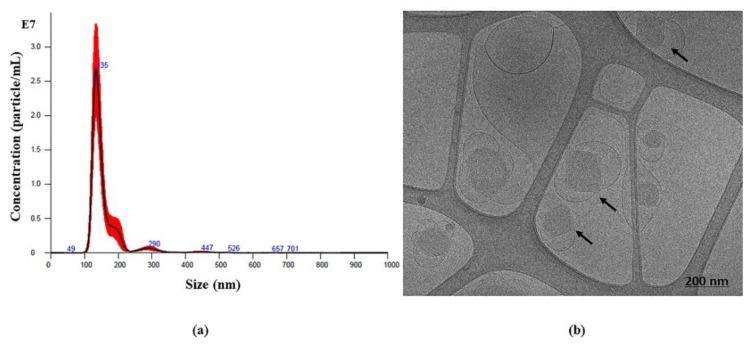
(**a**) Nanoparticle tracking analysis of GaPP-LCNP presented from five consecutive 60 s runs. The graph indicates monodispersity of nanoparticles with mode value of 135 nm. (**b**) Cryo-TEM image of GaPP-LCNP showing cubic shaped lyotropic liquid crystals (cubosomes) with particle average diameter of 150 nm.

To confirm the monodispersity of GaPP-LCNP, we used nanoparticle tracking analysis; the mean diameter of GaPP-LCNP was 156 nm with narrow size distribution, as shown in Figure 2a. Furthermore, cryo-transmission electron microscopy (Cryo-TEM) images, as shown in Figure 2b, demonstrated the morphology of LCNP to be cubic in shape (cubosomes), in addition to the presence of some vesicles, which are formed during the dilution process and deemed to be cubosomes’ precursors [22], which eventually undergo phase transition to form cubosomes [16]. 

Moreover, the successful entrapment of GaPP within LCNP bilayer has modulated its optical properties, evidenced by higher molar absorption coefficient (ɛ) with GaPP-LCNP 57983 M^−1^cm^−1^, compared to GaPP in 1% DMSO solution 19689 M^−1^cm^−1^. The absorption coefficient is an important factor in determining the density of photons absorbed by photosensitizers [23]; higher ɛ indicates better photodynamic activity as lower photosensitizer concentrations and light dose may be needed to achieve the photoactivation process [24]. 

**Figure 3:**
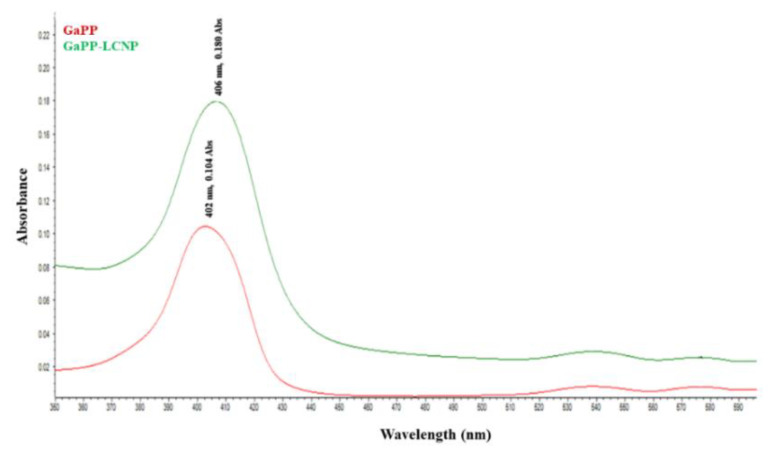
Absorption spectra of GaPP (1.5 µM) dissolved in 1% DMSO solution (red line) and GaPP-LCNP (1.5 µM) (green line).

The enhanced photodynamic activity of GaPP within LCNP is ascribed to the presence of GaPP in freely soluble monomer form, which is characterized by absorption maxima at 406 nm (Soret band) and 539 nm (Q band) [25]. The visible spectrum of GaPP-LCNP indicated that GaPP within LCNP mostly occurs in monomer form with higher absorption maxima at 406 nm and 539 nm, compared to unformulated GaPP that showed lower absorption peaks with slight blue shift at 402 nm, as shown in Figure 3. GaPP can exist in three forms in solution, either as freely soluble monomer (GaPP), a dimer (GaPP)_2_ associated with a single H_2_O adduct, or a trimer (GaPP)_3_, which is adducted by two water molecules [25]. The occurrence of H_2_O molecules with dimer and trimer GaPP indicates aggregation, as H_2_O is sandwiched between the tetrapyrrole rings of GaPP [25].

Occurrence of dimers and trimers reduces π–π* transition of electrons [25], which lowers the photodynamic activity of GaPP [26]. In addition to shielding GaPP from interactions with H_2_O, the architecture of the LCNP lipid bilayer prevents the aggregation of GaPP molecules within the nanoparticles via π–π interaction, which has been reported in liposomes due to the smaller surface area of the lipid bilayer [27,28]. These data correlate with our previous findings of higher singlet oxygen (^1^O_2_) quantum yield (ɸ) of GaPP-LCNP 0.72, compared to 0.42 with unformulated GaPP solution [8].

### 3.2. Antibacterial Photodynamic Activity against Planktonic Culture

**Figure 4:**
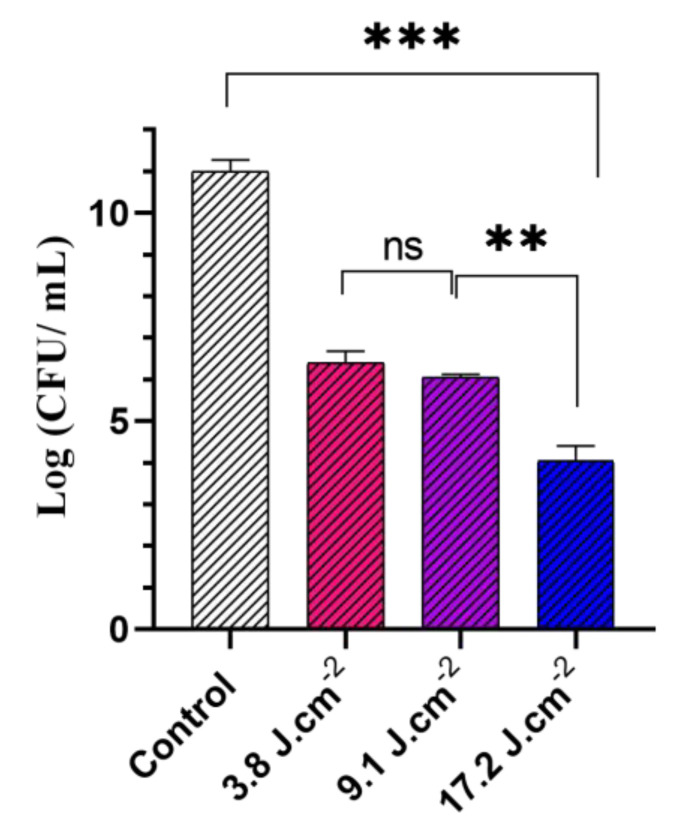
The viability of *P. aeruginosa* planktonic culture following treatment with different light doses of blue light at 405 nm ranging from (3.8 to 17.2) J.cm^−2^ and GaPP-LCNP concentration of 1.5 µM compared to negative control in the dark. Data presented as mean ± SD, *n* = 3. ns: non-significant, *** significant reduction in viability, *p* value < 0.0001, ** *p* value = 0.003 (one-way ANOVA test followed by multiple comparison Tukey’s test).

Key parameters affecting the photodynamic activity of GaPP-LCNP against *P. aeruginosa* were optimized to achieve maximum bacterial inactivation, namely light dose, GaPP concentration, and incubation time. Light dose is a detrimental factor for aPDT activity [29]; therefore, we tested different light doses between (3.8 to 17.2 J.cm^−2^) at 405 nm to determine the optimum light dose for inactivation of planktonic culture Figure 4. Maximum antibacterial activity was achieved at a light dose of 17.2 J.cm^−2^, where the viability of *P. aeruginosa* culture was reduced by ~7 log_10_. In general, Gram-negative bacteria are more tolerant to photoinactivation due to the presence of the outer lipopolysaccharide membrane that provides extra protection to the cell [3]. Light doses between (20–50 J.cm^−2^) have been reported for photoinactivation of *P. aeruginosa* using renowned photosensitizers, e.g., methylene blue [30].

**Figure 5:**
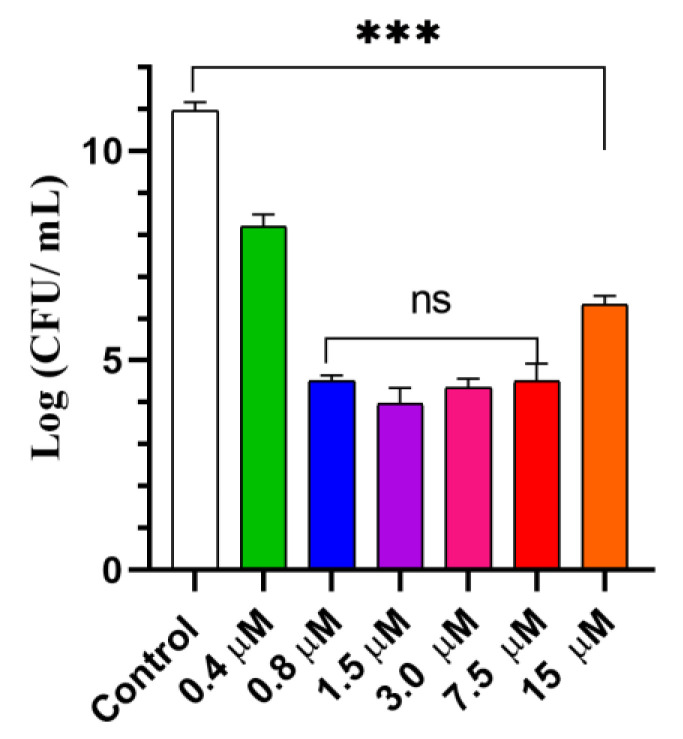
The viability of *P. aeruginosa* planktonic culture following treatment with different concentrations of GaPP-LCNP (0.4 to15 µM), light dose of 17.2 J.cm^−2^ compared to negative control treated with saline in the dark. Data presented as mean ± SD, *n* = 3. ns: non-significant, *** significant reduction in viability, *p* value < 0.0001, ** *p* value = 0.003 (one-way ANOVA test followed by multiple comparison Tukey’s test).

Following light dose optimization, a series of GaPP concentrations in LCNP ranged from (0.4 to 15 µM) were investigated to determine the highest antibacterial activity. A total of 0.8 µM GaPP in LCNP reduced the viability of *P. aeruginosa* by ~7 log_10_, while further increase in GaPP concentration did not significantly improve the antibacterial activity, rather a decline was recorded at 15 µM concentration Figure 5. 

This decline in the antibacterial activity at higher GaPP concentration can be ascribed to the photobleaching of GaPP by the generated ^1^O_2_, a phenomenon that has been reported with other porphyrin derivative photosensitizer, e.g., Foscan^®^ [31]. Atif et al. reported that at constant light fluence there was a direct correlation between photosensitizer concentration and photobleaching [32]. This was attributed to the interaction between the higher concentrations of generated ^1^O_2_ and photosensitizers molecules at ground state, leading to its degradation. To correlate the lower antibacterial activity with photobleaching, we determined the fluorescence intensities (FI) of GaPP at two concentrations, 1.5 µM and 15 µM, both in solution and in LCNP before and after illumination for 5 min, 0.057 W/cm^2^.

Higher photobleaching was recorded with GaPP-LCNP with 97% and 90% reduction in FI at 15 µM and 1.5 µM, respectively, compared to 88% and 72 % reduction with unformulated GaPP solution at the same concentrations. The photobleaching effect was more pronounced in GaPP-LCNP due to the fact GaPP-LCNP produced higher ^1^O_2_ with quantum yield (ɸ_Δ_) = 0.72, compared to unformulated GaPP ɸ_Δ_ = 0.42 [8], which correlates with the findings of Atif et al. [32] that higher ^1^O_2_ concentrations are responsible for the photobleaching phenomenon. These findings are essential for adequate dose optimization of photosensitizer concentration and the influence of formulation before judging its antimicrobial activity.

**Figure 6:**
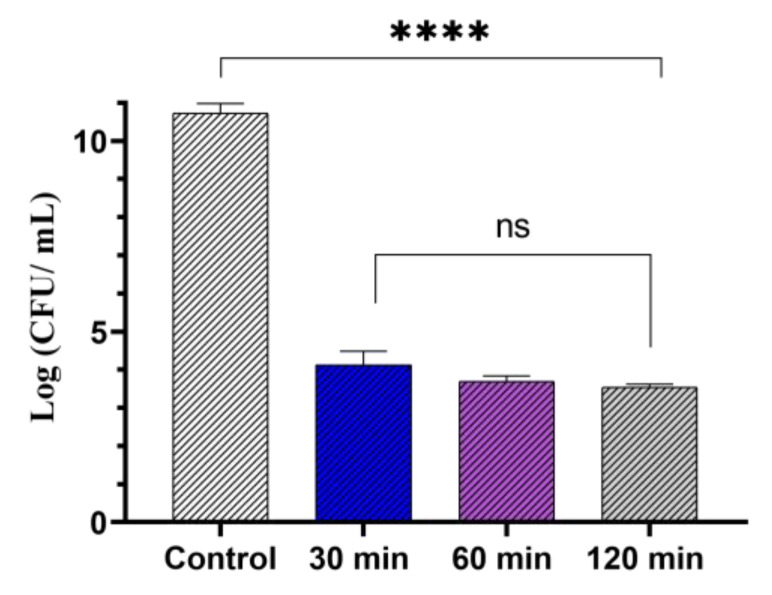
The viability of *P. aeruginosa* planktonic culture following treatment with GaPP-LCNP (0.8 µM), light dose of 17.2 J.cm^−2^, compared to negative control after 2 h in the dark. Data presented as mean ± SD, *n* = 3. ns: non-significant reduction, **** significant reduction *p* value < 0.0001 (one-way ANOVA test followed by Tukey’s multiple comparison test).

The third parameter that was optimized was pre-incubation time. GaPP-LCNP were incubated with *P. aeruginosa* in the dark for different periods of time before photoinactivation and the viability was assessed using CFU enumeration. A total of 30 min pre-incubation time was necessary to reduce the viability of *P. aeruginosa* by 7 log_10_; photoactivation at shorter incubation periods (5–15 min) did not reduce the viability of *P. aeruginosa* (data not presented), while longer incubation periods up to 120 min did not further reduce the viability of bacterial culture, as shown in Figure 6. 

Incubating photosensitizers with bacterial cultures before illumination, is essential to achieve significant antibacterial activity, particularly Gram-negative strains, due to the nature of Gram-negative cell walls, which requires longer incubation times for photosensitizers to diffuse through it to reach cell membrane [30]. It was previously reported that incubation for 12 h was necessary to achieve 3.5 log_10_ reduction in the viability of *P. aeruginosa* using chitosan nanoparticles loaded with Erythrosine at a light dose of 50 J.cm^−2^ [33]. However, in our study, 30 min was sufficient for GaPP-LCNP to inactivate *P. aeruginosa*, which is ascribed to the fusion uptake mechanism of LCNP, overcoming *P. aeruginosa* outer membrane [34]. An experimental visualization of LCNP interaction with *E. coli* cell walls has demonstrated that LCNP attached to the cell walls within 30 min followed by complete diffusion of its cargo to bacterial cytoplasm within ~ 90 min [35], which correlates with our findings that 30 min were necessary for LCNP to attach to *P. aeruginosa* cell walls, allowing the generated ^1^O_2_ to disrupt *P. aeruginosa* cell walls.

**Figure 7:**
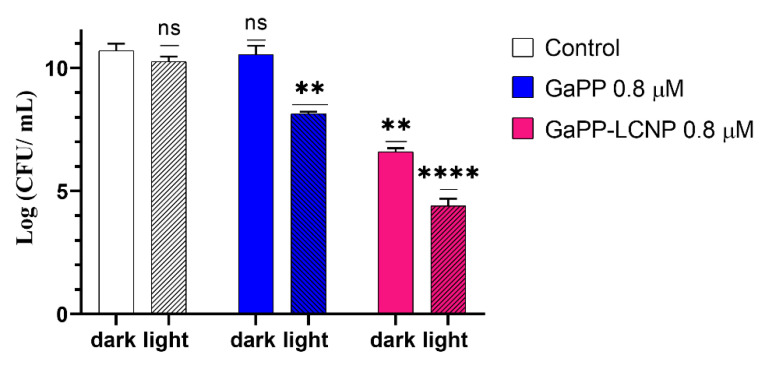
The viability of *P. aeruginosa* planktonic culture following treatment with GaPP and GaPP-LCNP (0.8 µM), light dose of 17.2 J/cm^2^, compared to saline treated control in dark. Data presented as mean ± SD, *n* = 3. ns: non-significant reduction, **** significant reduction in viability, *p* value < 0.0001, ** *p* value = 0.001 (Two-way ANOVA followed by Dunnett’s multiple comparison test).

The enhanced delivery of GaPP to *P. aeruginosa* via LCNP through this fusion mechanism [34,35] has significantly enhanced its antibacterial activity both as an iron mimic agent and photosensitizer reducing the viability of *P. aeruginosa* by 4 log_10_ in the dark and 6.5 log_10_ upon light activation Figure 7. On the other hand, unformulated GaPP solution did not show any antibacterial activity as iron mimic agent as *P. aeruginosa* acquires iron through the siderophore pyoverdine rather than the heme acquisition system [36]. While as photosensitizer GaPP reduced the viability of *P. aeruginosa* by only 2.5 log_10_. The lower activity of GaPP, compared to GaPP-LCNP, is ascribed to the inability of hydrophobic GaPP molecules to diffuse through a *Pseudomonas* outer cell wall that limits the diffusion of the hydrophobic compounds [37]. These findings differ from our previous study against *S. aureus* [8], where GaPP antibacterial activity as an iron mimetic agent was more profound, reducing the viability of *S. aureus* by 4 log_10_ in the dark and completely eradicating bacterial colonies at the same concentration and much lower light dose (0.8 J/cm^2^). The higher antibacterial activity towards *S. aureus* is attributed to their iron uptake mechanism through hemophores [11], which allows the inactivation of *S. aureus* via disrupting their metabolic activity in the dark and maximizes the damaging action of generated ROS upon light activation [8].

### 3.3. Antibacterial Activity against Biofilms 

**Figure 8:**
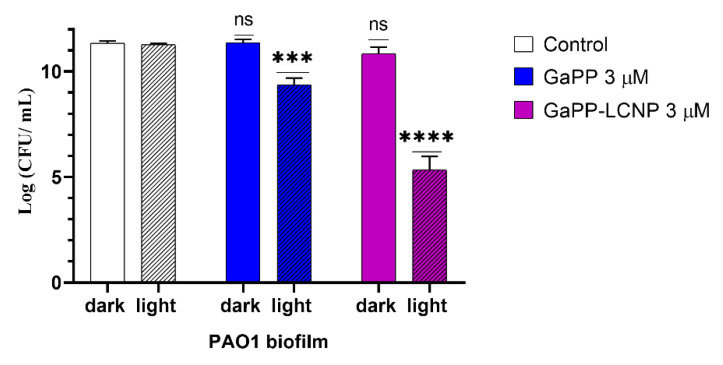
The viability of *P. aeruginosa* biofilm following treatment with GaPP and GaPP-LCNP (3 µM), light dose of 34.2 J/cm^2^, compared to saline treated control in dark. Data presented as mean ± SD, *n* = 3. ns: non-significant reduction, *** significant reduction *p* value = 0.0007, **** *p* = <0.0001, (Two-way ANOVA, followed by multiple comparison Dunnett’s test).

In general, biofilms are more tolerant to antimicrobial treatments, including aPDT, than planktonic bacteria due to the protective nature of EPS [38]. The biofilm matrix of *P. aeruginosa* is a key factor in its resistance to antimicrobials [14]. Two major contributors in biofilm matrix are the polysaccharides Pel and Psl [39] that give the biofilm its 3D structure and repel the diffusion of hydrophobic compounds, such as GaPP [34]. Therefore, disrupting biofilm matrix is an effective approach to combat biofilm resistance and inactivate bacterial cells within biofilms [40]. In this study, the effective aPDT conditions used for inactivation of planktonic culture, i.e., 0.8 µM GaPP and 17.2 J.cm^−2^, did not induce antibacterial activity to *P. aeruginosa* biofilms. However, rising the light dose to 34.2 J.cm^2^, after incubation with 3 µM GaPP in the dark for 2 h significantly reduced the viability of biofilms by 2 log_10_ and 6 log_10_ for GaPP and GaPP-LCNP, respectively, as shown in Figure 8.

**Figure 9:**
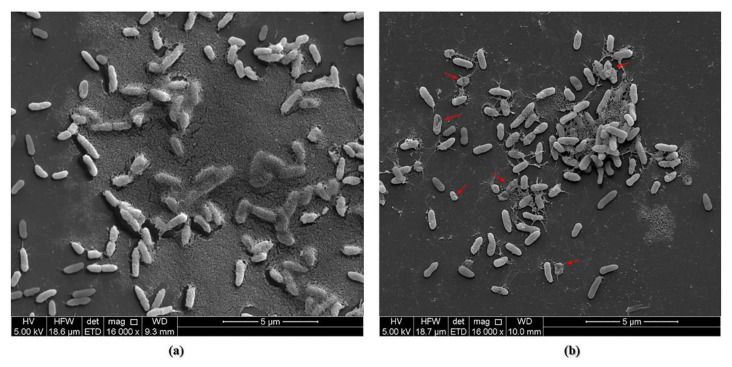
(**a**) SEM images of *P. aeruginosa* PAO1 biofilms demonstrates biofilm matrix covering large portions of the bacterial cells in negative controls sample, (**b**) SEM image representative of biofilms observed after treatment with GaPP-LCNP 3 µM and a light dose of 34.2 J/cm^2^. The biofilm matrix was disrupted, and the morphology of bacterial cell walls changed, presumably by the action of ROS.

**Figure 10:**
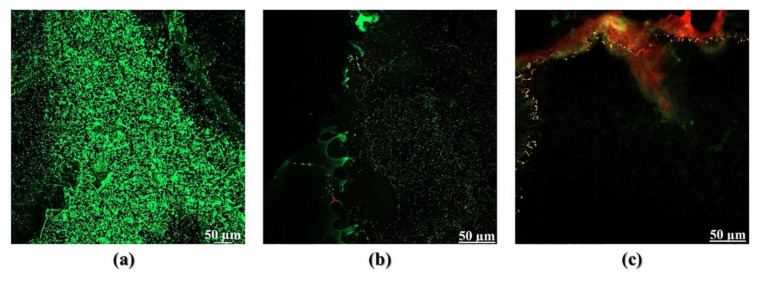
(**a**) Confocal images of *P. aeruginosa* PAO1 biofilms stained with Syto-9 (green) live stain and Propidium iodide (red) dead stain in negative control sample, (**b**) PAO1 biofilm incubated with 3 µM GaPP for 2 h and photoactivated with 34.2 J.cm^−2^, (**c**) PAO1 biofilm incubated with 3 µM GaPP-LCNP for 2 h and photoactivated with 34.2 J.cm^−2^.

The higher antibacterial activity of GaPP-LCNP, compared to unformulated GaPP, is attributed to the higher ROS production [8] and the ability of LCNP to attach to biofilms forming a coating patch on biofilm surface [34]. The attachment of LCNP to biofilms has maximized the damaging effect of the generated ROS by disrupting the biofilm matrix and inactivating embedded bacterial cells in the biofilm, as illustrated by SEM images in Figure 9.The confocal images have given further insights to the superior activity of GaPP-LCNP over unformulated GaPP, as the killing effect of the generated ROS was extended beyond the boundaries of biofilm, reaching the embedded bacterial cells, while unformulated GaPP showed moderate antibacterial activity localized on the biofilm surface, as shown in Figure 10.

**Figure 11:**
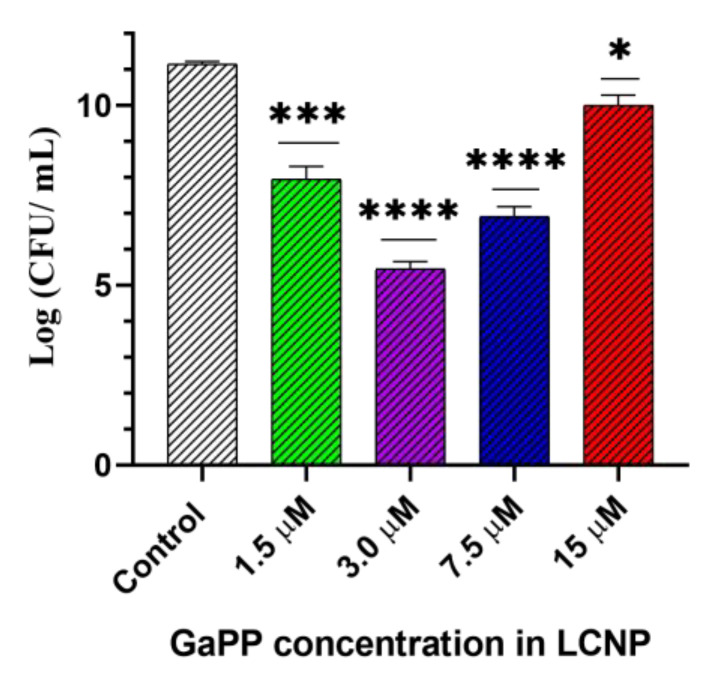
Viability of *P. aeruginosa* biofilm following treatment with different concentrations of GaPP-LCNP, light dose 34.2 J/cm^2^, compared to saline control in the dark. Data presented as mean ± SD, *n* = 3. Significant reduction was calculated using one-way ANOVA test followed by Dunnett’s multiple comparison test. * *p* = 0.026, *** *p* = 0.0002, **** *p* < 0.0001.

Akin to our findings with planktonic culture, increasing GaPP concentrations above an optimum value (3 µM) was accompanied with a decline in the antibacterial activity due to the photobleaching phenomenon Figure 11. The photobleaching of GaPP-LCNP in biofilms is not only due to the higher quantum yield of ^1^O_2_ generated from GaPP-LCNP but also to the spatial distribution of GaPP in biofilms [41]. Photobleaching theories established in the studies that were conducted to dose porphyrin-based photosensitizers to cancer cells [41,42] concluded that a wide distribution of photosensitizer molecules within target cells maximizes the chances of the generated ^1^O_2_ to interact with other molecular targets before interacting with photosensitizer molecules [42]. Similarly, the distribution of photosensitizer molecules within biofilm matrix determines the degree of photobleaching. In another study conducted by our lab (data not published) on *S. aureus* biofilms, we found that the photobleaching effect on GaPP-LCNP was negligible due to their wide distribution within biofilm matrix. This wide distribution was not only due to the fusion ability of LCNP but also to the hunger of *S. aureus* to GaPP as haem mimetic [8].

Yet, in the case of *P. aeruginosa* biofilms, LCNP forms a coating patch around biofilm [34], and the uptake of GaPP is limited due to the different iron acquisition mechanism utilized through siderophores [36], which maximizes the probability of destroying adjacent GaPP molecules by generated ^1^O_2_. Neither the increase of illumination time nor the pre-incubation period improved the antibacterial activity of GaPP-LCNP, as GaPP molecules are photobleached after 10 min of photoactivation. Increasing the pre-incubation time beyond 2 h was not beneficial, as LCNP crystalline structure was disrupted via digestion of monooleate by *Pseudomonas* lipase [43]. The digestion takes about 2 h, as previously determined during the release study of GaPP from LCNP [8]. Although the digestion of LCNP by bacterial lipase is considered a beneficial approach to facilitate the uptake of antimicrobial peptides by *P. aeruginosa* biofilms [44], it is not as favorable for the photoactivation of GaPP as the integrity of LCNP is disrupted, which was found to play a role in maximizing ROS production upon photoactivation [8].

These findings strengthen our claim of using GaPP-LCNP as a third-generation photosensitizer against *Pseudomonas* biofilms that offer remarkable antibacterial activity with high safety profile. The treatment conditions used against biofilms (3 µM GaPP-LCNP, 34.2 J/cm^2^) were proven to be highly safe to human fibroblasts; we previously investigated the safety of a series of GaPP-LCNP concentrations on human fibroblasts and no reduction in viability was noticed up to 30 µM GaPP-LCNP [8]. Furthermore, using blue light at 405 nm showed no adverse effects on human fibroblasts up to 36 J/cm^2^ [45], which is higher than the light dose used in our study, confirming the safety of our approach to human skin and the potential application of GaPP-LCNP to control *P. aeruginosa* biofilms associated with superficial infections. In contrast, higher light doses of 428.5, 322, and 483 J/cm^2^ were reported to activate methylene blue, a renowned photosensitizer for antimicrobial applications [30,46]; however, these studies could not achieve similar antibiofilm activity even with high energy fluences.

Several factors contribute to the higher antibacterial activity of GaPP-LCNP. Firstly, the liquid crystalline structure of the nanoparticles that protect GaPP from aggregating in biological media [8], maximizing GaPP light absorption and ROS production. Secondly, the attachment of LCNP to *Pseudomonas* biofilms forming a coating patch [34] that helps overcome ^1^O_2_ limitations of short lifetime and short diffusion length (3.5 µs and ~100 nm) [47]. Thirdly, despite their negative charge, LCNP can alter *P. aeruginosa* cell wall permeability via quick fusion with bacterial outer membranes, such as cationic entities [48]. All these mechanisms combined qualifies GaPP-LCNP to be a promising photosensitizer against resistant localized infections.

## 4. Conclusions

In this study, GaPP-LCNP has shown superior photodynamic activity against the notorious Gram-negative bacterium *P. aeruginosa*, reducing the viability of planktonic culture by 7 log_10_. Furthermore, GaPP-LCNP reduced the viability of biofilms 500 times, compared to unformulated GaPP, and successfully disrupted biofilm matrix at a relatively lower light dose and photosensitizer concentration, compared to reported approaches. The lower GaPP-LCNP dose utilized in this study elaborate both high activity and safety profile of GaPP-LCNP, enabling the inactivation of bacterial biofilms without adversely affecting host tissues [8]. Future studies against *P. aeruginosa* clinical isolates would be worthwhile to ascertain the wide spectrum antibacterial activity of GaPP-LCNP against multi-drug resistant bacteria. Furthermore, the success of GaPP-LCNP in disrupting biofilm matrix justifies investigating the potential synergy between GaPP-LCNP and antibiotics to completely eradicate *P. aeruginosa* within biofilms to avoid infection recurrence.

## Figures and Tables

**Table 1:** Physicochemical characteristics of LCNP formulations.

Sample	Mean Particle Size (nm)	Z-Average Diameter (nm)	Polydispersity Index (PDI)	Zeta Potential (mV)	EE%	DL *w*/*w*%
Blank LCNP	175 ± 2.2	184 ± 2.7	0.18 ± 0.04	−24.4 ± 0.71		
GaPP-LCNP	156 ± 1.4	175 ± 3.4	0.21 ± 0.02	−29.9 ± 0.91	98% ± 3.0	3.3 ± 0.3

## Data Availability

Not applicable.
